# Bmal1 Downregulation Worsens Critical Limb Ischemia by Promoting Inflammation and Impairing Angiogenesis

**DOI:** 10.3389/fcvm.2021.712903

**Published:** 2021-08-10

**Authors:** Lirong Xu, Yutong Liu, Qianyun Cheng, Yang Shen, Ye Yuan, Xiaolang Jiang, Xu Li, Daqiao Guo, Junhao Jiang, Changpo Lin

**Affiliations:** ^1^Department of Pathology, School of Basic Medical Science, Shanghai University of Traditional Chinese Medicine, Shanghai, China; ^2^Department of Physiology and Pathophysiology, School of Basic Medical Science, Fudan University, Shanghai, China; ^3^Department of Vascular Surgery, Institute of Vascular Surgery, Zhongshan Hospital, Fudan University, Shanghai, China

**Keywords:** circadian clock, Bmal1, critical limb ischemia, lipids metabolism, inflammation, angiogenesis

## Abstract

Critical limb ischemia (CLI) is the most advanced clinical stage of peripheral vascular disease with high mobility and mortality. CLI patients suffer from lower extremity rest pain, ulceration, and gangrene caused by insufficient blood and oxygen supply. Seeking for effective biomarkers and therapeutic targets is of great significance for improving the life quality of CLI patients. The circadian clock has been reported to be involved in the progression of kinds of cardiovascular diseases. Whether and how circadian genes play a role in CLI remains unknown. In this study, by collecting femoral artery and muscle specimens of CLI patients who underwent amputation, we confirmed that the circadian gene Bmal1 is downregulated in the CLI femoral artery and ischemic distal lower limb muscle. Furthermore, we verified that Bmal1 affects CLI by regulating lipid metabolism, inflammation, and angiogenesis. A hindlimb ischemia model performed in wild-type and Bmal1^−/−^ mice confirmed that Bmal1 disruption would lead to impaired angiogenesis. *In vitro* experiments indicated that the decreased expression of Bmal1 would increase ox-LDL uptake and impair endothelial cell functions, including proliferation, migration, and tube formation. As for mechanisms, Bmal1 represses inflammation by inhibiting lipid uptake and by activating IL-10 transcription and promotes angiogenesis by transcriptionally regulating VEGF expression. In conclusion, we provide evidence that the circadian gene Bmal1 plays an important role in CLI by inhibiting inflammation and promoting angiogenesis. Thus, Bmal1 may be an effective biomarker and a potential therapeutic target in CLI.

## Introduction

Critical limb ischemia (CLI) is an ischemic disease of the lower extremities caused by arterial stenosis and occlusion (1). It is a local manifestation of systemic atherosclerosis in the limbs. Its pathological progress starts with arterial intima and middle layer degenerative and proliferative changes and then leads to arterial wall thickening, hardening, and twisting, resulting in arterial lumen stenosis and even obstruction and ultimately leading to the occurrence of corresponding ischemic symptoms at the distal end of the artery (2). There are numerous theories about the CLI etiology, including lipid deposition, inflammation, and thrombosis (2, 3). Kinds of inflammatory factors and cytokines are involved in inflammatory injury, plaque formation, plaque rupture, and final artery stenosis or occlusion (4, 5). Patients with peripheral arterial diseases often have accompanying kinds of severe comorbidities, including coronary artery disease, cerebrovascular disease, respiratory dysfunction, and end-stage renal disease (6). Thus, CLI seriously damages the health of patients, and more importantly it is strongly related to a high risk of mortality, especially due to cardiovascular events. Therefore, it is urgent to clarify the detailed pathogenesis of CLI and seek out more efficient prediction and therapy methods.

Disordered lipid metabolism has been recognized as the independent risk factor of atherosclerosis and related peripheral vascular disease (7). High levels of circulating LDL or modified LDL (i.e., ox-LDL) induce endothelial cell (EC) dysfunctions and increased adhesion molecule and proinflammatory gene expression (8–10). Accordingly, LDL and ox-LDL are the major causes of atherosclerotic lesions (10). LDL would deposit at the vascular walls, with a high serum concentration (8). ox-LDL binds to several kinds of scavenger receptors located at macrophages and ECs, including CD36, macrophage scavenger receptor 1 (MSR-1), and lectin-like oxidized low-density lipoprotein receptor 1 (LOX-1) (10). Among these, LOX-1 is the main ox-LDL receptor in ECs and mediates a host of ox-LDL-induced effects (8, 10). Once modified LDL stimulates the ECs, circulating monocytes are recruited through the activated endothelium and differentiate into macrophages (11). The activated macrophages produce inflammatory cytokines, chemokines, oxygen and nitrogen radicals, and other inflammatory molecules, ultimately leading to inflammation and tissue damage (11). The important inflammatory molecules in this process include interferon-γ, interleukin-1 (IL-1), interleukin-6 (IL-6), and tumor necrosis factor α (TNFα) (11). On the other hand, anti-inflammatory factors, including interleukin-10 (IL-10) and transforming growth factor b (TGF-β), act as protective factors in atherosclerosis. The inhibition of IL-10 (12, 13) and TGF-β (14) aggravates atherosclerosis. The balance between inflammation and anti-inflammation controls the progression of atherosclerosis and related peripheral vascular diseases. However, the role of lipid metabolism and inflammation in the progression of CLI is still unclear, and the relevant mechanism needs further investigation.

Angiogenesis occurs in response to arterial occlusion and shear force changes to restore blood flow and nutrient supply in the heart and limbs (15). This is of great significance in the treatment of ischemic diseases caused by arterial occlusion. The vascular endothelial growth factor (VEGF) plays a critical role in angiogenesis by activating target angiogenesis-related genes (15). A hypoxic environment, inflammation-related cytokines, and hormones are reported to be able to elevate the expression of VEGF (15, 16). However, the VEGF expression is attenuated in the aged and hypercholesterolemic ones. It is urgent to restore angiogenesis to cure CLI and other vascular obstruction diseases in these kinds of people (17).

The circadian clock, as a comprehensive regulation system that controls the wake–sleep cycle, body temperature, hormone secretion, etc. of an organism, plays a pivotal role in the metabolic regulation process (18). Disordered circadian rhythms would inevitably lead to severe metabolic disorders and related diseases (19–22). Studies have shown that people with circadian clock disorders are more likely to suffer from cardiovascular diseases, metabolic-related diseases, and cancer (23–26). It is reported that the occurrence of acute arterial occlusion of the limbs showed a significant circadian pattern with a peak in the early morning (27). Mice with knockout or mutant circadian clock genes are accompanied with an abnormal activity rhythm, metabolic disorders, and cardiovascular diseases (20, 22, 28). As for Bmal1, the core circadian gene, it plays a critical role in lipid metabolism, inflammation, and related cardiovascular diseases. It has been reported that plasma cholesterol ester, non-esterified fatty acids, and phospholipids are all elevated in Bmal1^−/−^ mice compared with wild-type (WT) mice of the same age (22). Besides this, Bmal1 deficiency affects the cholesterol efflux to the bile. Thus, a global Bmal1 deficiency increases atherosclerosis (22). Moreover, an organ-specific knockout of Bmal1 would also lead to disordered lipid metabolism and atherosclerosis, including the liver (22), endothelial cells (29), and myeloid cells (30). Among them, mice with liver Bmal1 deficiency have accompanying increased hepatic triglyceride and cholesterol levels (22). Moreover, myeloid Bmal1 deficiency leads to proinflammatory macrophage phenotype changes and enhances monocyte recruitment to the atherosclerotic lesion, which then aggravates atherosclerosis (30). However, the role of circadian genes, especially Bmal1, in CLI needs more investigations.

This research aims to explore the relationship between the circadian gene Bmal1 and CLI. By collecting ischemic artery and the lower limb muscle of CLI patients, constructing a lower limb ischemia animal model, and conducting *in vitro* experiments in endothelial cells, we demonstrated that the disruption of Bmal1 aggravates lipid deposition and inflammation and impairs angiogenesis. Therefore, the downregulation of Bmal1 would promote the occurrence and progression of CLI. Our research may help to find effective serum markers for the early diagnosis and prevention of CLI and to develop new potential therapeutic targets.

## Materials and Methods

### Patients

With the approval of the Ethical Committee of Zhongshan Hospital, three pairs of femoral artery tissues and normal artery specimens from healthy donors were collected. Nine groups of lower limb muscle were obtained from CLI patients with lower limb amputations. All patients signed the informed consent before enrollment in the study. The study was conducted in accordance with the ethical guidelines of the Declaration of Helsinki.

### Animals

Bmal1^+/−^ mice are introduced from the Jackson Laboratory and had been bred in the Model Animal Research Center of Nanjing University (The GemPharmatech Company). Heterozygous mice were intercrossed to obtain homozygous Bmal1-deficient (Bmal1^−/−^) mice as well as control wild-type mice. All mice were fed with a chow diet and raised in a clean room with 12-h light and 12-h dark cycles. All animal experiments were conducted strictly in accordance with the National Institutes of Health Guide for the Care and Use of Laboratory Animals and were approved by the Animal Care and Use Committee of Shanghai Medical College, Fudan University.

### Cell Culture

Human umbilical vein endothelial cells (HUVECs) were obtained from ATCC. The HUVECs were grown in 1640 medium supplemented with 10% fetal bovine serum (FBS), 10 U/ml penicillin and 100 mg/ml streptomycin at 37°C with 5% CO_2_.

The 293T cells were introduced from the Cell Bank Type Culture Collection of the Chinese Academy of Sciences. They were cultured in Dulbecco's modified Eagle's medium (DMEM) medium supplemented with 10% FBS, 10 U/ml penicillin, and 100 mg/ml streptomycin. The cells were cultured in a humidified CO_2_ incubator at 37°C.

### Oil Red O Staining

To determine the lipid deposition in the sub-endothelial femoral artery, femoral arteries from CLI patients and normal arteries are fixed with 4% paraformaldehyde and then frozen-sectioned and stained with Oil Red O.

### HLI Mouse Model

At 6–8 weeks of age, the mice were anesthetized with pentobarbital sodium (50 mg/kg, i.p.) and then subjected to unilateral femoral artery ligation and resection. The blood flow in the lower limb was monitored with a laser Doppler perfusion imaging system (Perimed, Inc., Ardmore, PA) immediately after surgery (day 0) and then at days 7 and 14 post-surgery. The mice were placed on a warming pad during surgery and during laser Doppler image acquisition to maintain a constant body temperature of 37°C. Perfusion was expressed as the ratio of the left (ischemic) to right (non-ischemic) hindlimb. The right hindlimb served as an internal control for each mouse.

### Small Interfering RNA, Vector, and Lentivirus Infection

The siRNAs used to silence BMAL1 expression (siBMAL1), VEGF expression (si-VEGF), and negative control siRNA (si-NC) were designed and produced by RiboBio company (Shanghai, China) which were transfected into HUVECs by Lipofectamine® 3000 (Invitrogen; Thermo Fisher Scientific, Inc.) following the manufacturer's instruction after the cell density reached 80% confluence. To perform lentiviral infection, we allowed the cells to reach 70% confluence. During the infection, the medium was replaced with fresh medium containing lentivirus (ad-BAML1 and ad-GFP), and the cells were cultured for 24 h at 37°C, the culture was changed with a fresh complete medium, and the cells were continuously cultured for another 24 h.

### OX-LDL Treatment

A total of 40 μg/ml 1,1′-dioctadecyl-3,3,3′,3′-tetramethyl-indocarbocyanine perchlorate (Dil)-ox-LDL was added to the culture medium of HUVECs for 6 h. For confocal microscopy, the cells were fixed with 4% formaldehyde in room temperature for 15 min and visualized using standard rhodamine excitation: emission filters at 554:571 nm. For gene expression detection, the cells were harvested with the TRIzol reagent according to the instruction manual.

### Cell Proliferation Assay

A total of 2 × 10^4^ HUVECs were seeded on a 12-well plate (Thermo) and incubated in culture medium for 96 h at 37°C and 5% CO_2_. The cells in each well were digested with trypsin and counted at 24, 48, 72, and 96 h. Each experiment was repeated three independent times.

### Cell Migration Assay

The HUVECs (6 × 10^4^) were placed into Transwell chambers (Corning Incorporated, USA) for the migration assay. The lower chambers were filled with DMEM containing 10% FBS as a chemoattractant. After maintaining at 37°C for 6–8 h, the cells that remained on the upper surface of the membrane were removed. The HUVECs on the lower surface of the membrane were fixed with 4% formaldehyde and stained with 0.1% crystal violet. The stained cells were photographed and quantified by counting in five random microscopic fields.

### Tube Formation Assay

A total of 1 × 10^4^ HUVECs were suspended in culture medium and seeded in 48-well plates that were pretreated with Matrigel matrix. The formation of the tube networks develops in 12 h at 37°C and 10% CO_2_ and were visualized by a microscope and photographed at 3, 6, 8, and 12 h, and the analysis was performed with Image J. Each experiment was repeated three independent times.

### Reverse Transcription-Quantitative Polymerase Chain Reaction

Total RNA was isolated from tissues and cells using the TRIzol reagent (Invitrogen; Thermo Fisher Scientific, Inc.) following the protocol of the supplier. To detect mRNA expression, total RNA was reverse-transcribed into cDNA using a ReverTra Ace® qPCR RT Kit (code no. FSQ-201, Toyobo, Japan), and real-time PCR was performed using a SYBR Green kit (Toyobo, Japan). All samples were analyzed using a Roche real-time analyzer, and the results were normalized to the glyceraldehyde-3-phosphate dehydrogenase (GAPDH) expression. The primers used are listed in Supplementary Table 1.

### Western Blotting

Cultured cells or tissues were lysed using the RIPA lysis buffer, and total protein concentration was detected using bicinchoninic acid assay (Beyotime Institute of Biotechnology). SDS–PAGE (10%) was used to resolve equal amounts of protein. The membranes were blocked with 5% milk in PBS–Tween-20 buffer (PBST) for 1 h at room temperature prior to overnight incubation at 4°C with primary antibodies against specific antibodies (listed in Supplementary Table 2). Subsequently, the membranes were peroxidase-conjugated with secondary antibodies at room temperature for 1.5 h. After three rinses with PBST, the protein bands were visualized using the ECL western blotting substrate (Bio-Rad). β-ACTIN and GAPDH were considered the loading control for normalization.

### Chromatin Immunoprecipitation Assay

A total of 1 × 10^7^ HUVEC were washed with cold phosphate-buffered saline, fixed with 1% formaldehyde for 10 min at room temperature, and then crosslinked with 125 mM glycine for 5 min at room temperature. The cells were then harvested in cell lysis buffer (50 mM HEPES, 500 mM NaCl, 1% Triton X-100, 0.1% sodium deoxycholate, 1 mM EDTA, and 0.1% SDS, pH 7.5). The cell lysates were then briefly sonicated to fragment genomic DNA. The cell lysates were then used for chromatin immunoprecipitation using anti-BMAL1 and anti-IgG antibodies. The protein A/G beads–antibody/chromatin complexes were washed with lysis buffer and wash buffer (50 mM Tris-HCl, 300 mM LiCl, 2 mM MgCl, and 0.5% NP-40, pH 7.5), and the antibody/chromatin complexes were subsequently eluted with the elution buffer (50 mM Tris-HCl, 10 mM EDTA, and 1% SDS, pH 8.0). The cross-linked protein/DNA complexes were detached at 65°C for 4 h, followed by purification of the genomic DNA. The PCR primers are shown in Supplementary Table 1.

### Luciferase Reporter Assay

Luciferase reporter assay was conducted with the Firefly Luciferase Reporter Gene Assay Kit from Beyotime company. The primers for the luciferase reporter constructs are listed in Supplementary Table 3.

### Statistical Analysis

Data are presented as the mean ± SEM. Statistical comparisons were conducted with unpaired Student's *t*-tests/one-way ANOVA with *post-hoc* Tukey test/two-way ANOVA with *post-hoc* Sidak test as appropriate, and *p* < 0.05 was considered statistically significant.

## Results

### Disruption of Bmal1 Expression Is Associated With Critical Limb Ischemia

**Figure 1:**
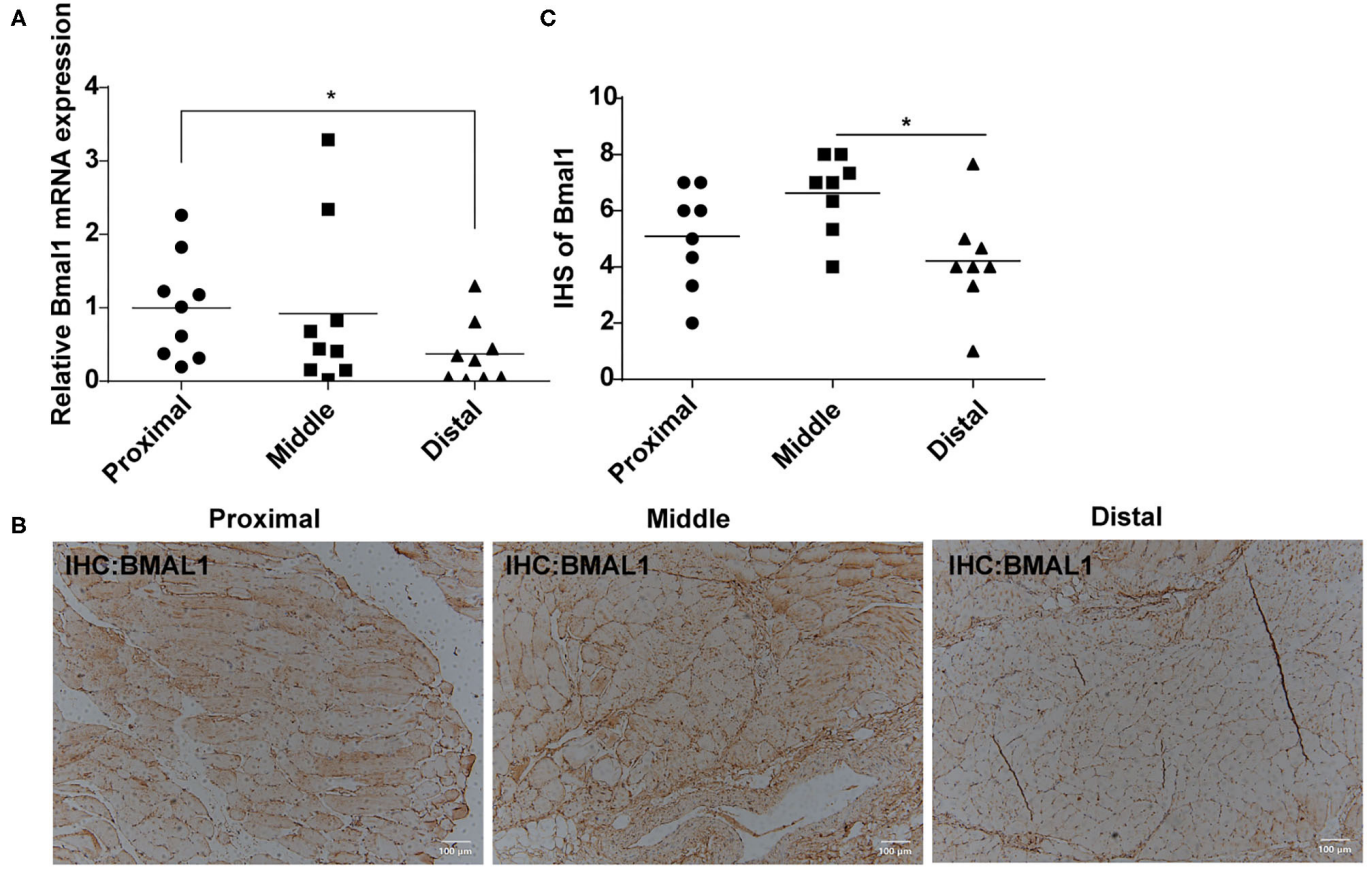
Bmal1 expression is decreased in the distal lower limb muscle of critical limb ischemia (CLI) patients. **(A)** Relative mRNA expression of Bmal1 in the lower limb muscle of CLI patients detected by real-time PCR. Data are presented as mean ± SEM (*n* = 9, one-way ANOVA with *post-hoc* Tukey test). ^*^p < 0.05 distal lower limb vs. proximal lower limb. **(B,C)** Immunohistochemistry conducted in the lower limb muscle of CLI patients with specific anti-BMAL1 antibody with a dilution of 1:100. The immunohistochemical staining results were assigned with a mean score considering the intensity of staining and the proportion of cells with a positive reaction area. Each section was independently assessed by two pathologists without prior knowledge of patient data. Data are presented as mean ± SEM (*n* = 8, one-way ANOVA with *post-hoc* Tukey test). ^*^p < 0.05 distal lower limb vs. middle lower limb.

Considering the close relationship between circadian clock and cardiovascular diseases, we were wondering whether Bmal1 plays a role in critical limb ischemia. First of all, we compared the Bmal1 expression differences in femoral artery specimens from critical limb ischemia (CLI) patients and normal artery specimens from healthy donors (NA). It was shown that there is a significant decreased expression of Bmal1 in CLI femoral artery specimens compared with the normal artery (Supplementary Figure 1A), suggesting that Bmal1 may play a protective role in the CLI occurrence and progression. To further investigate the relationship between Bmal1 and CLI progression, we collected the proximal, middle, and distal lower limb muscle of CLI patients with different levels of ischemic severity. The demographic characteristics of these patients are shown in Supplementary Table 4. The mRNA (Figure 1A) and protein (Figures 1B,C) expressions of Bmal1 are both decreased in the distal lower limb muscle compared with the proximal, while there was a slight increase of BMAL1 protein expression in the middle lower limb muscle compared with the proximal. These results suggested that the decreased Bmal1 expression may promote the progression of CLI and aggravate the ischemic symptoms of the lower extremities.

### Bmal1 Inhibits Inflammation by Repressing Lipid Uptake and Activating IL-10 Expression

**Figure 2:**
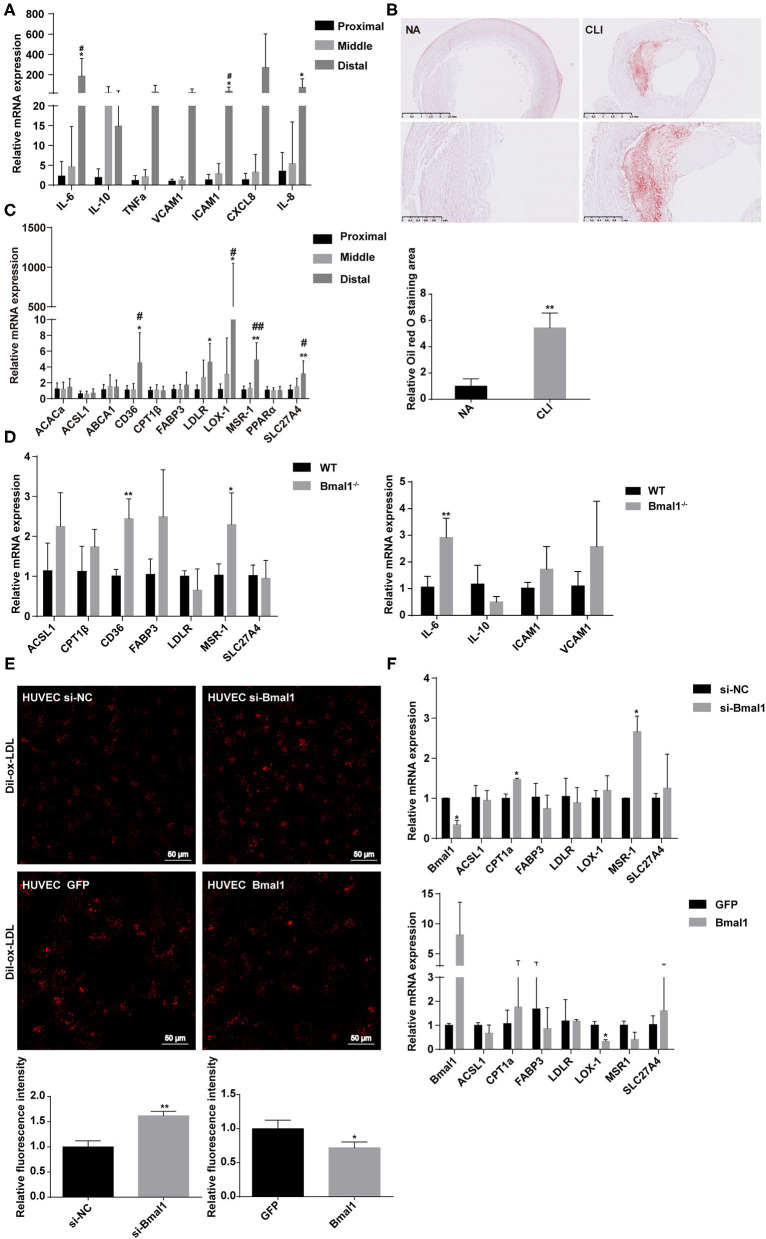
Bmal1 inhibits lipid uptake by endothelial cells. **(A)** Relative mRNA expression of inflammation factors by real-time PCR. Data are presented as mean ± SEM (*n* = 9, one-way ANOVA with *post-hoc* Tukey test). ^*^p < 0.05 distal lower limb vs. proximal lower limb; ^#^*p* < 0.05 distal lower limb vs. middle lower limb. **(B)** Oil red O staining of normal artery and femoral artery from critical limb ischemia (CLI) patients. Oil red O staining area are measured with Image J. Data are presented as mean ± SEM (*n* = 3, unpaired *t*-test); ^**^p < 0.01 CLI vs. NA. **(C)** Relative mRNA expression of genes involved in lipid metabolism in the lower limb muscle of CLI patients. Data are presented as mean ± SEM (*n* = 9, one way ANOVA with *post-hoc* Tukey test). ^*^p < 0.05 and ^**^p < 0.01, distal lower limb vs. proximal lower limb; ^#^*p* < 0.05 and ^##^*p* < 0.01, distal lower limb vs. middle lower limb. **(D)** Relative mRNA expression of genes involved in lipid metabolism and inflammation in wild-type and Bmal1^−/−^ mice lower limb muscle. Data are presented as mean ± SEM (*n* = 4, unpaired *t*-test). ^*^p < 0.05 and ^**^p < 0.01, Bmal1^−/−^ vs. WT. **(E)** Dil-ox-LDL uptake by human umbilical vein endothelial cells (HUVECs), with the expression changes of Bmal1 measured by confocal microscopy. Ox-LDL uptake is measured with the relative fluorescence intensity. Data are presented as mean ± SEM (unpaired *t*-test, repeated 3 independent times). ^**^p < 0.01 si-Bmal1 vs. si-NC and ^*^p < 0.05 Bmal1 vs. GFP. **(F)** Relative mRNA expression of genes involved in lipid metabolism in HUVECs with the expression changes of Bmal1. Data are presented as mean ± SEM (unpaired *t*-test, repeated 3 independent times). ^*^p < 0.05, si-Bmal1 vs. si-NC; ^*^p < 0.05 Bmal1 vs. GFP.

Although CLI can be caused by vasculitis, thromboembolism, trauma, and Buerger disease, it is mostly associated with atherosclerosis (3). Atherosclerosis is a chronic disease closely related to inflammation, which is initiated by an inflammatory response resulting in elevated lipid deposition, EC dysfunction, and monocyte recruitment to the arterial intima (31). We wondered whether Bmal1 plays a role in CLI progression by affecting inflammation and lipid metabolism. First of all, we explored the expression of inflammatory factors in CLI patients. The mRNA expression of genes involved in inflammation is elevated in the femoral artery specimens of CLI patients, including pro-inflammation factors like IL-6 and TNFα and anti-inflammation factor IL-10 (Supplementary Figure 2A). The inflammation factor expressions were both increased in the middle and distal muscle of the lower limbs compared with the proximal lower limb muscle, while the increase in distal group is much more obvious, suggesting that inflammation is more serious in the distal lower limb (Figure 2A). By oil red O staining, we then verified that the sub-endothelial deposition of lipids was more pronounced in the femoral artery of CLI patients (Figure 2B). We then explored the mRNA expression of genes involved in lipid metabolism, including fatty acid and cholesterol uptake, transportation, and metabolism in the femoral artery and lower limb muscle of CLI patients. It was shown that low-density lipoprotein receptor and ox-LDL scavenger receptors, including CD36 and LOX-1, were elevated in CLI femoral artery (Supplementary Figure 2B), suggesting more lipid uptake in CLI patients. Furthermore, the above-mentioned gene expression was also increased in the distal lower limb muscles compared with the proximal ones (Figure 2C), indicating the association of lipid uptake with the severity of ischemia.

To further investigate the role of Bmal1 in these processes, we then explored these gene expressions in WT and Bmal1^−/−^ mice lower limb muscle and found a significant increase of CD36, MSR-1, and IL-6 in Bmal1^−/−^ mice (Figure 2D). Moreover, we measured the plasma lipid profiles in Bmal1^−/−^ mice and their littermates of WT mice. It was shown that the triglyceride and LDL-C content are elevated in Bmal1^−/−^ mice, while the HDL-C level is downregulated in Bmal1^−/−^ mice compared with the WT mice (Supplementary Figure 3A). These results suggested that Bmal1 may inhibit the uptake of lipids by endothelial cells and macrophages by inhibiting ox-LDL receptors, thereby repressing inflammation and playing a protective role in the occurrence of CLI. To prove our hypothesis, we knocked down and overexpressed Bmal1 expression in HUVECs and treated the cells with Dil-labeled ox-LDL. When Dil-ox-LDL was taken up by vascular endothelial cells or macrophages, the lipoprotein is degraded by lysosomal enzymes and the Dil (fluorescent probe) accumulates in the intracellular membranes. It was shown that knocking down of Bmal1 in HUVECs caused an increase of Dil-ox-LDL uptake, while the overexpression of Bmal1 was associated with a downregulation of Dil-ox-LDL uptake (Figure 2E). Besides this, the mRNA expression of ox-LDL receptors, including MSR-1 and LOX-1, was consistent with the ox-LDL uptake result (Figure 2F). Therefore, our results demonstrated that Bmal1 inhibits inflammation in CLI by inhibiting the lipid uptake of endothelial cells.

**Figure 3:**
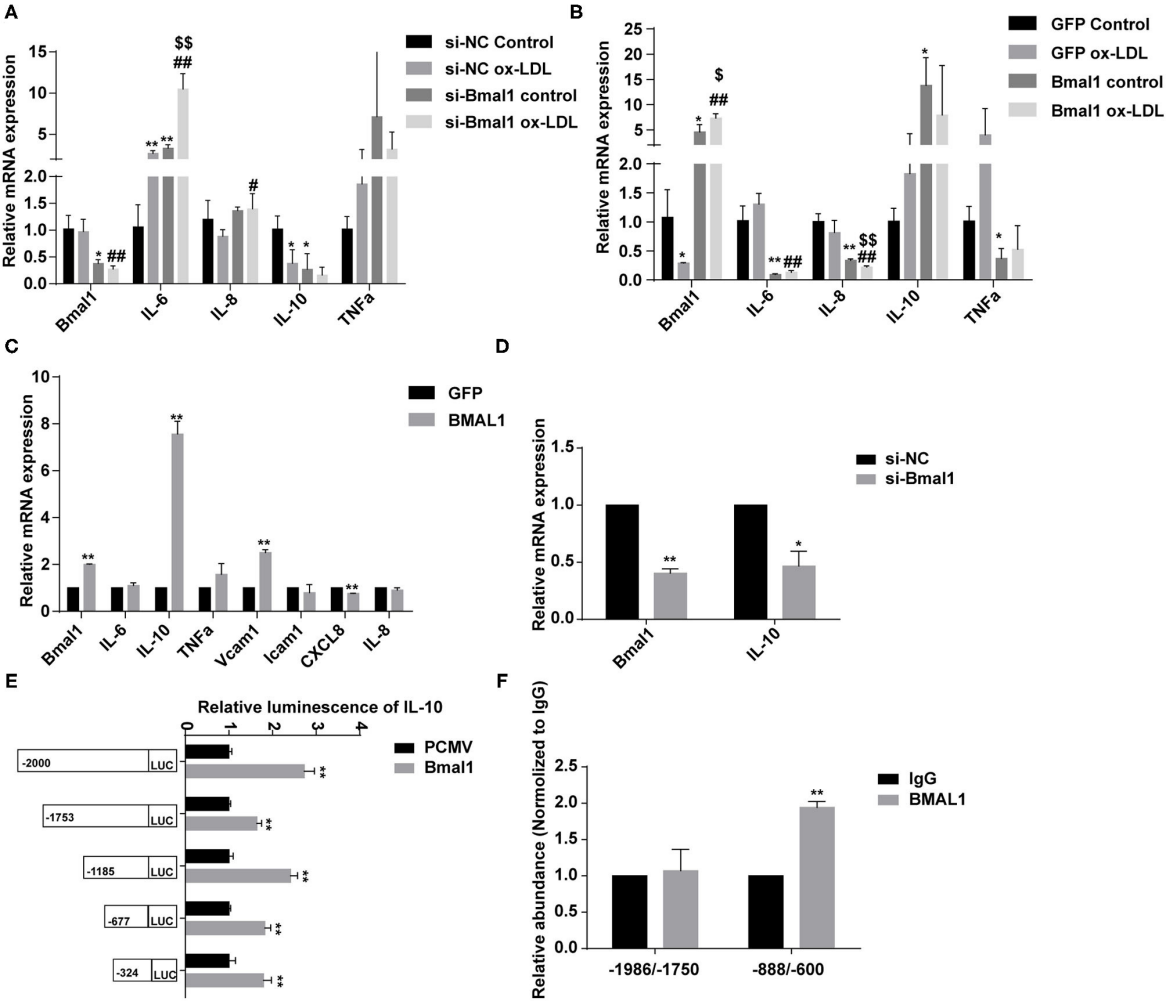
Bmal1 represses inflammation by inhibiting the lipid uptake and transcriptionally regulating the IL-10 expression. **(A)** Relative mRNA expression of genes involved in inflammation in human umbilical vein endothelial cells (HUVECs) which had been transfected with si-NC or si-Bmal1 with or without the addition of ox-LDL. Data are presented as mean ± SEM (one-way ANOVA with *post-hoc* Tukey test). ^*^p < 0.05 and ^**^p < 0.01 vs. si-NC control; ^#^*p* < 0.05 and ^##^*p* < 0.01 vs. si-NC ox-LDL; ^$$^*p* < 0.01 vs. si-Bmal1 control. **(B)** Relative mRNA expression of genes involved in inflammation in HUVECs which had been transfected with adenoviruses coding for AdGFP or AdBmal1 with or without the addition of ox-LDL. Data are presented as mean ± SEM (one-way ANOVA with *post-hoc* Tukey test). ^*^p < 0.05 and ^**^p < 0.01 vs. GFP control; ^##^*p* < 0.01 vs. GFP ox-LDL; ^$^*p* < 0.05 and ^$$^*p* < 0.01 vs. Bmal1 control. **(C)** Relative mRNA expression of genes involved in inflammation in HUVECs which had been transfected with adenoviruses coding for AdGFP or AdBmal1. Data are presented as mean ± SEM (unpaired *t*-test). ^**^p < 0.01 Bmal1 vs. GFP. **(D)** Relative mRNA expression of Bmal1 and IL-10 in HUVECs which had been transfected with Bmal1 siRNA or si-NC. Data are presented as mean ± SEM (unpaired *t*-test). ^*^p < 0.05 and ^**^p < 0.01 si-Bmal1 vs. si-NC. **(E)** Luciferase reporter constructs were created containing the truncated (−2,000, −1,753, −1,185, −677, and −324) versions of the IL-10 promoter. The luciferase reporter constructs were co-transfected with Bmal1 overexpression vector or with the control vector into HEK293T cells, and luciferase activity was evaluated 24 h later. Data are presented as mean ± SEM (unpaired *t*-test). ^**^p < 0.01 Bmal1 vs. PCMV. **(F)** ChIP assay conducted in HUVECs with anti-BMAL1 or IgG antibody. qRT-PCR analysis was performed with primer sequences around Bmal1-binding E-box elements in the IL-10 promoter. Data are presented as mean ± SEM (unpaired *t*-test). ^**^p < 0.01 BMAL1 vs. IgG. Each expreiment was repeated 3 independent times.

To further demonstrate that Bmal1 regulates inflammation by affecting the lipid uptake, we changed the expression of Bmal1 together with the addition of ox-LDL. The expression alterations of the inflammatory genes verified that the addition of ox-LDL would increase the pro-inflammatory gene expression and decrease the anti-inflammatory gene expression. Besides this, the knockdown of Bmal1 would aggravate the changes, while the overexpression of Bmal1 would alleviate these changes (Figures 3A,B). Furthermore, we wondered whether Bmal1 can directly regulate inflammation. By overexpressing and knocking down Bmal1, we found that Bmal1 positively regulates IL-10 expression (Figures 3C,D). Moreover, luciferase reporter assay and chromatin immunoprecipitation (ChIP) assay indicated that Bmal1 transcriptionally regulates IL-10 expression by binding on its promoter region (Figures 3E,F). In conclusion, we demonstrated that Bmal1 represses inflammation in CLI by inhibiting lipid deposition and promoting the anti-inflammatory factor IL-10 expression.

### Disruption of Bmal1 Impairs Angiogenesis in CLI

**Figure 4:**
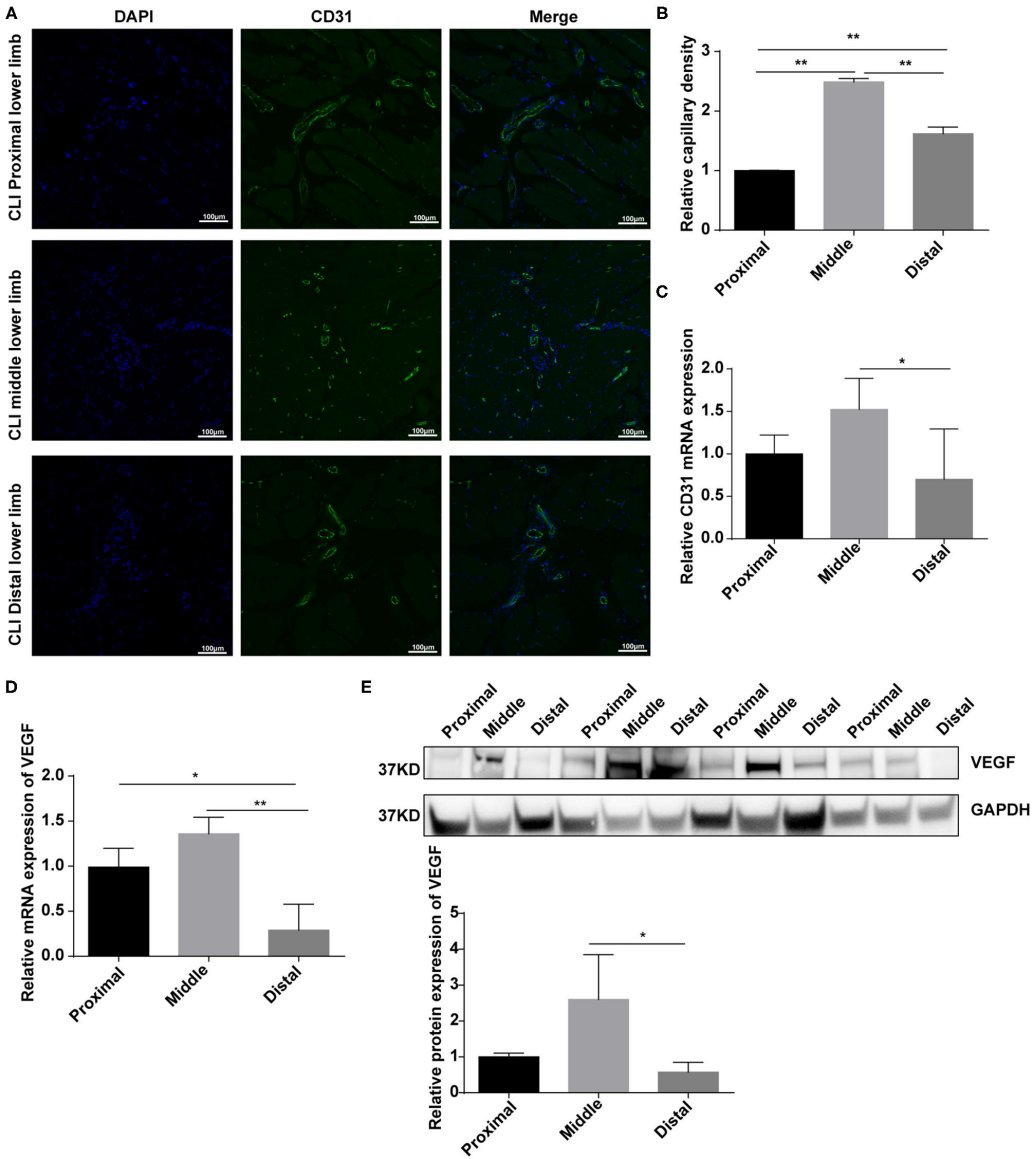
Angiogenesis are elevated in response to vascular occlusion in the lower limb muscle of critical limb ischemia (CLI) patients. **(A,B)** Immunofluorescence conducted in the lower limb muscle of CLI patients with specific anti-CD31 antibody. Relative capillary density was determined based on immunofluorescence staining. Data are presented as mean ± SEM (*n* = 9, one-way ANOVA with *post-hoc* Tukey test). ^**^p < 0.01, middle lower limb vs. proximal lower limb; ^**^p < 0.01, distal lower limb vs. proximal lower limb; ^**^p < 0.01, distal lower limb vs. middle lower limb. **(C)** Relative mRNA expression of CD31 in the lower limb muscle of CLI patients by real-time PCR. Data are presented as mean ± SEM (*n* = 9, one-way ANOVA with *post-hoc* Tukey test). ^*^p < 0.05 middle lower limb vs. proximal lower limb; ^*^p < 0.05 distal lower limb vs. middle lower limb. **(D)** Relative mRNA expression of VEGF in the lower limb muscle of CLI patients by real-time PCR. Data are presented as mean ± SEM (*n* = 9, one-way ANOVA with *post-hoc* Tukey test). ^**^p < 0.01 distal lower limb vs. middle lower limb; ^*^p < 0.05 distal lower limb vs. proximal lower limb. **(E)** Relative protein expression of VEGF in the lower limb muscle of CLI patients by western blot (*n* = 4, one-way ANOVA with *post-hoc* Tukey test). Data are presented as mean ± SEM.

Angiogenesis stimulated by vascular occlusion helps restore blood and oxygen supply to the lower limbs and further alleviate symptoms. We then aimed to explore the role of Bmal1 in this process. By real-time PCR, we found that the mRNA expression of genes involved in angiogenesis is elevated in the femoral artery of CLI patients (Supplementary Figure 4A). We then explored the difference of angiogenesis in the proximal, middle, and distal lower limb muscle. The immunofluorescence of CD31, an endothelial cell marker, suggested that the capillary density is much more abundant in the middle than the distal lower limb (Figures 4A,B). Moreover, the mRNA expression of CD31 increased significantly in the middle lower limb muscle, suggesting that angiogenesis is more announced (Figure 4C). However, there was no significant increase in the distal group despite of more serious ischemia (Figure 4C). We then wondered whether the decreased angiogenesis was related to BMAL1, CD31, and BMAL1 immunofluorescence co-staining results which showed that the BMAL1 expression was reduced in the endothelial cells in the distal lower limb muscle of CLI patients (Supplementary Figures 5A,B). We then detected the expression of VEGF, which is the critical factor in angiogenesis, and found that it was decreased significantly in the distal group (Figures 4D,E). These results indicated that decreased angiogenesis leads to serious ischemic symptoms in the distal lower limb muscle, and it is partly attributed to reduced VEGF expression.

**Figure 5:**
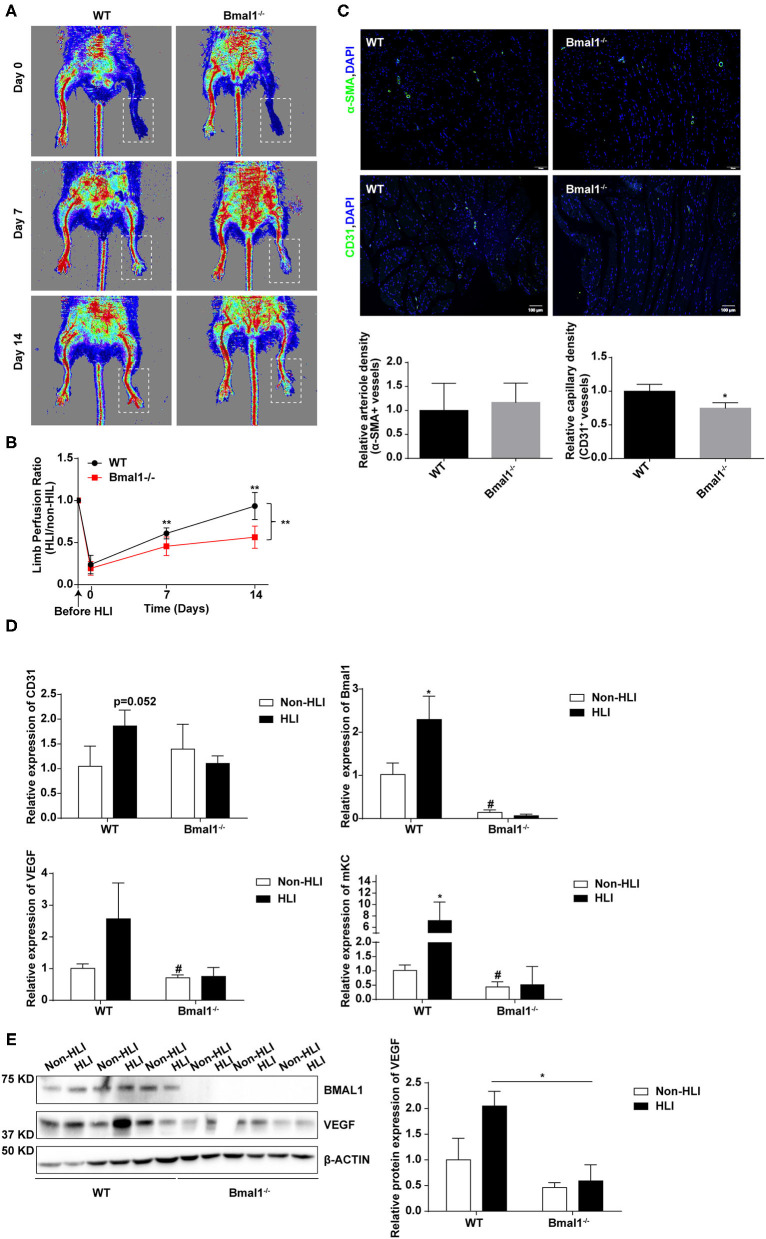
Bmal1 promotes angiogenesis after peripheral ischemic injury. **(A)** Blood flow obtained *via* laser Doppler perfusion imaging on days 0, 7, and 14 after HLI in Bmal1^−/−^ mice and WT mice. **(B)** The perfusion of the hindlimbs of mice at each time point was calculated as the ratio of measurements of the injured (HLI) and uninjured (non-HLI) limbs. *n* = 4 for Bmal1^−/−^ and WT mice. Data are presented as mean ± SEM (unpaired *t*-test and two-way ANOVA with *post-hoc* Sidak test). ^**^P < 0.01 Bmal1^−/−^ vs. WT. **(C)** At 14 days after HLI, the gastrocnemius muscle was harvested from the HLI limb of Bmal1^−/−^ mice and WT mice and stained for α-smooth muscle actin (α-SMA) and CD31 expression. Data are presented as mean ± SEM (*n* = 4, unpaired *t*-test). ^*^P < 0.05 Bmal1^−/−^ vs. WT. **(D)** mRNA levels of the CD31, Bmal1, VEGF, and murine functional IL-8 homolog keratinocyte-derived chemokine (mKC) were evaluated in HLI and non-HLI limbs *via* real-time PCR. Data are presented as mean ± SEM (*n* = 4, unpaired *t*-test). ^*^p < 0.05 wild-type (WT) HLI vs. WT non-HLI; ^#^*p* < 0.05 Bmal1^−/−^ non- HLI vs. WT non-HLI. **(E)** Protein expression level of BAML1 and VEGF in HLI and non-HLI limbs measured by western blot. Data are presented as mean ± SEM (*n* = 3, unpaired *t*-test). ^*^p < 0.05 Bmal1^−/−^ HLI vs. WT HLI.

To determine whether Bmal1 affects CLI by regulating angiogenesis, hindlimb ischemia (HLI) was surgically performed in Bmal1^−/−^ mice and their littermates of WT mice. Blood flow measurements were performed at days 0, 7, and 14 after HLI. The results showed that, compared with the WT mice, the blood perfusion in the ischemic limbs of Bmal1^−/−^ mice was significantly inhibited (Figures 5A,B). Next, immunofluorescence staining of α-SMA and CD31 was performed in ischemic muscle collected 14 days after HLI. As shown in Figure 5C, a lower anti-CD31-positive capillary density was observed in Bmal1^−/−^ mice. Moreover, there is an obvious elevation of CD31 and α-SMA mRNA expression after HLI in WT mice, probably attributed to increased angiogenesis response after HLI (Figure 5D; Supplementary Figure 6A). However, the elevation effect was eliminated in Bmal1^−/−^ mice with a significantly decreased mRNA expression of α-SMA and CD31 in both non-HLI and HLI groups (Figure 5D; Supplementary Figure 6A). Moreover, Bmal1 expression was elevated in the WT HLI group compared with WT non-HLI group (Figures 5D,E). These results indicated the promoting effect of Bmal1 in angiogenesis. Consistently, Bmal1 deficiency was associated with a significant decrease in the expression of angiogenic factors, including mKC (a murine functional homolog of IL-8) and VEGF (Figures 5D,E). Moreover, the expression changes of inflammatory factors suggested that inflammation regulation in Bmal1^−/−^ mice is disordered (Supplementary Figure 6B). Therefore, we concluded that BMAL1 contributes to revascularization after ischemia in mice and CLI patients by promoting angiogenesis.

### Bmal1 Promotes the Angiogenic Activity of HUVECs by Transcriptionally Regulating VEGF Expression

**Figure 6:**
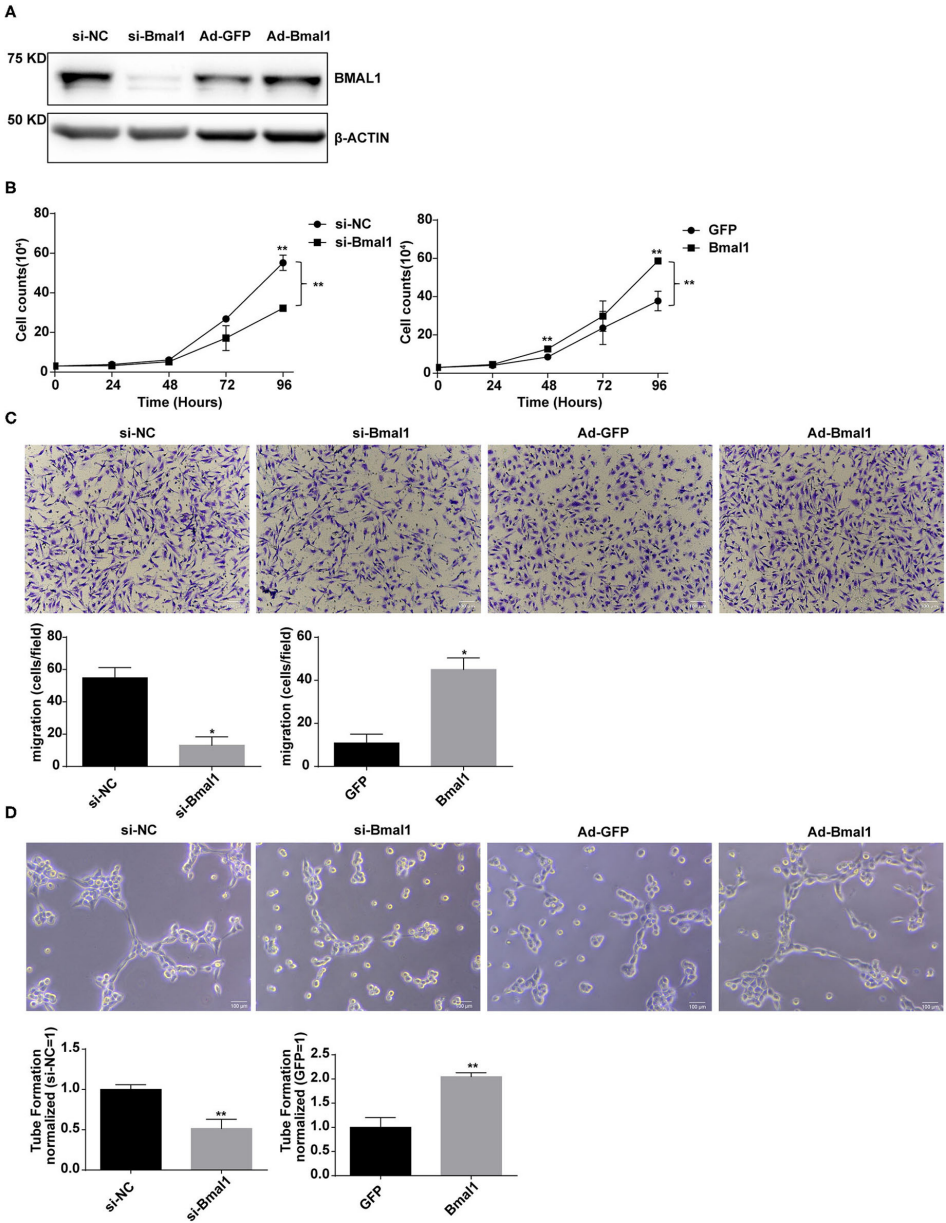
Bmal1 promotes the proangiogenic activity of endothelial cells. **(A)** Knockdown and overexpression effect of Bmal1 in human umbilical vein endothelial cells (HUVECs). Bmal1 protein levels were evaluated *via* western blotting assay. **(B)** Cell proliferation was measured by cell counting in HUVECs. Data are presented as mean ± SEM (unpaired *t*-test and two-way ANOVA with *post-hoc* Sidak test). ^**^p < 0.01 si-Bmal1 vs. si-NC; ^**^p < 0.01 Bmal1 vs. GFP. **(C)** Cell migration measured by transwell in HUVECs. Data are presented as mean ± SEM (unpaired *t*-test). ^*^p < 0.05 si-Bmal1 vs. si-NC; ^*^p < 0.05 Bmal1 vs. GFP. **(D)** Endothelial tube formation in matrigel in HUVECs. Data are presented as mean ± SEM (unpaired *t*-test). ^**^p < 0.01 si-Bmal1 vs. si-NC; ^**^p < 0.01 Bmal1 vs. GFP. Each expreiment was repeated 3 independent times.

To further determine the proangiogenic role of Bmal1, assessments of human umbilical vein cell (HUVEC) proliferation, migration, and tube formation were conducted in Bmal1 knocked down or overexpressed cells. The efficiency of Bmal1 knockdown and overexpression in HUVECs was confirmed by western blotting assay (Figure 6A). Cell proliferation in si-Bmal1 HUVEC was significantly slower than the control group (Figure 6B). Consistently, an opposite result was obtained in AdBmal1 HUVEC observed by cell counting assay (Figure 6B). Furthermore, transwell chamber experiment was conducted to determine the migration of HUVECs. The si-Bmal1 HUVEC showed impaired cell migration, while the AdBmal1-HUVEC significantly increased cell migration (Figure 6C). Moreover, compared with the control group, tube formation in Bmal1 siRNA-HUVEC was significantly reduced. In AdBmal1 HUVECs, tube formation was significantly promoted (Figure 6D). Since Bmal1 upregulation promoted HUVEC proliferation, migration, and tube formation, it is implicated that Bmal1 is involved in angiogenesis by maintaining HUVEC functions.

**Figure 7:**
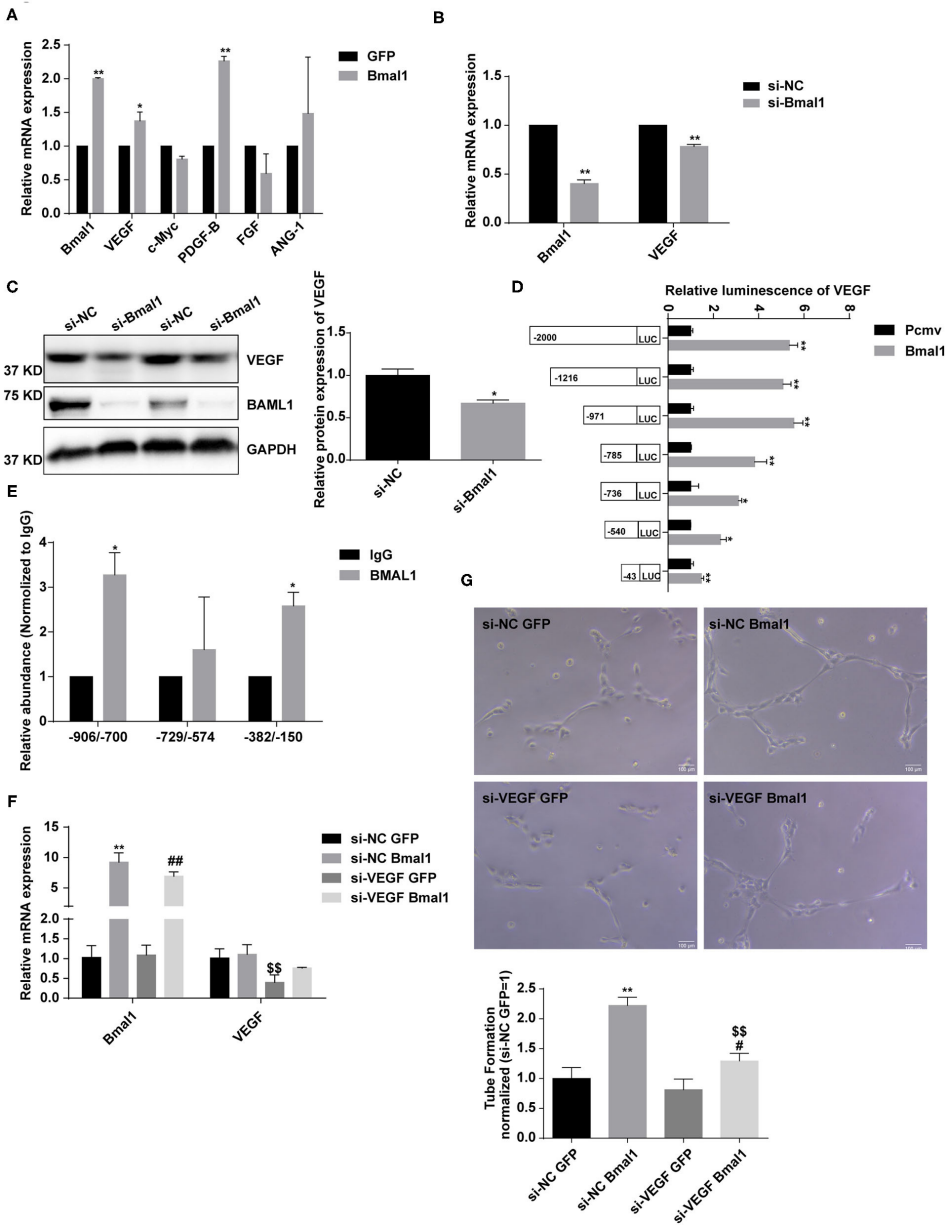
Bmal1 promotes angiogenesis by transcriptionally regulating the vascular endothelial growth factor (VEGF) expression. **(A)** Relative mRNA expression of genes involved in angiogenesis in human umbilical vein endothelial cells (HUVECs) which had been transfected with adenoviruses coding for AdGFP or AdBmal1. Data are presented as mean ± SEM (unpaired *t*-test). ^**^p < 0.01 Bmal1 vs. GFP; ^*^P < 0.05 Bmal1 vs. GFP. **(B,C)** Relative mRNA and protein expression of Bmal1 and VEGF in HUVECs which had been transfected with Bmal1 siRNA or si-NC. Data are presented as mean ± SEM (unpaired *t*-test). ^**^p < 0.01 si-Bmal1 vs. si-NC; ^*^p < 0.05 si-Bmal1 vs. si-NC. **(D)** Luciferase reporter constructs were created containing the truncated (−2,000, −1,216, −971, −785, −736, −540, and −43) versions of the VEGF promoter. The luciferase reporter constructs were co-transfected with Bmal1 overexpression vector or with the control vector into HEK293T cells, and luciferase activity was evaluated 24 h later. Data are presented as mean ± SEM (unpaired *t*-test). ^**^p < 0.01 and ^*^p < 0.05 Bmal1 vs. PCMV. **(E)** ChIP assay conducted in HUVECs with anti-BMAL1 or IgG antibody. A qRT-PCR analysis was performed with primer sequences around Bmal1-binding E-box elements in the VEGF promoter. Data are presented as mean ± SEM (unpaired *t*-test). ^*^p < 0.05 BMAL1 vs. IgG. **(F)** Relative mRNA expression of Bmal1 and VEGF in HUVECs which had been transfected with/without VEGF siRNA and Bmal1/GFP. Data are presented as mean ± SEM (one-way ANOVA with *post-hoc* Tukey test). ^**^p < 0.01 si-NC Bmal1 vs. si-NC GFP; ^##^*p* < 0.01 si-VEGF Bmal1 vs. si-VEGF GFP; ^$$^*p* < 0.01 si-VEGF GFP vs. si-NC GFP. **(G)** Endothelial tube formation in matrigel in HUVECs. Data are presented as mean ± SEM (one-way ANOVA with *post-hoc* Tukey test). ^**^p < 0.01 si-NC Bmal1 vs. si-NC GFP; ^#^*p* < 0.05 si-VEGF Bmal1 vs. si-VEGF GFP; ^$$^*p* < 0.01 si-VEGF Bmal1 vs. si-NC Bmal1. Each expreiment was repeated 3 independent times.

To further estimate the mechanism of Bmal1 in regulating angiogenesis, we overexpressed Bmal1 in HUVEC and examined the expression of genes involved in angiogenesis. We found that there is a significant increase of VEGF expression in the Bmal1 overexpressing HUVEC (Figure 7A). We then knocked down Bmal1 and found an obvious downregulation of VEGF (Figures 7B,C). Since Bmal1 is a transcription factor, we further investigated whether VEGF was under the transcriptional regulation of Bmal1 by luciferase reporter assay. We found that Bmal1 overexpression significantly increased the luciferase activity of VEGF promoter constructs (Figure 7D). Thus, we generated a series of luciferase reporter constructs containing the VEGF promoter and then investigated the effect of Bmal1 overexpression on the luciferase activity of these constructs. The result suggested that Bmal1 mainly binds on the −971 to 0 region of the VEGF promoter (Figure 7D). Furthermore, ChIP assay verified that Bmal1 binds on the −906 to −700 and −382 to −150 regions of VEGF promoter (Figure 7E). Besides this, knocking down of VEGF partly dismissed the pro-angiogenesis function of Bmal1 overexpression in HUVECs (Figures 7F,G). In conclusion, our results verified that Bmal1 promotes angiogenesis by transcriptionally regulating VEGF expression.

## Discussion

**Figure 8:**
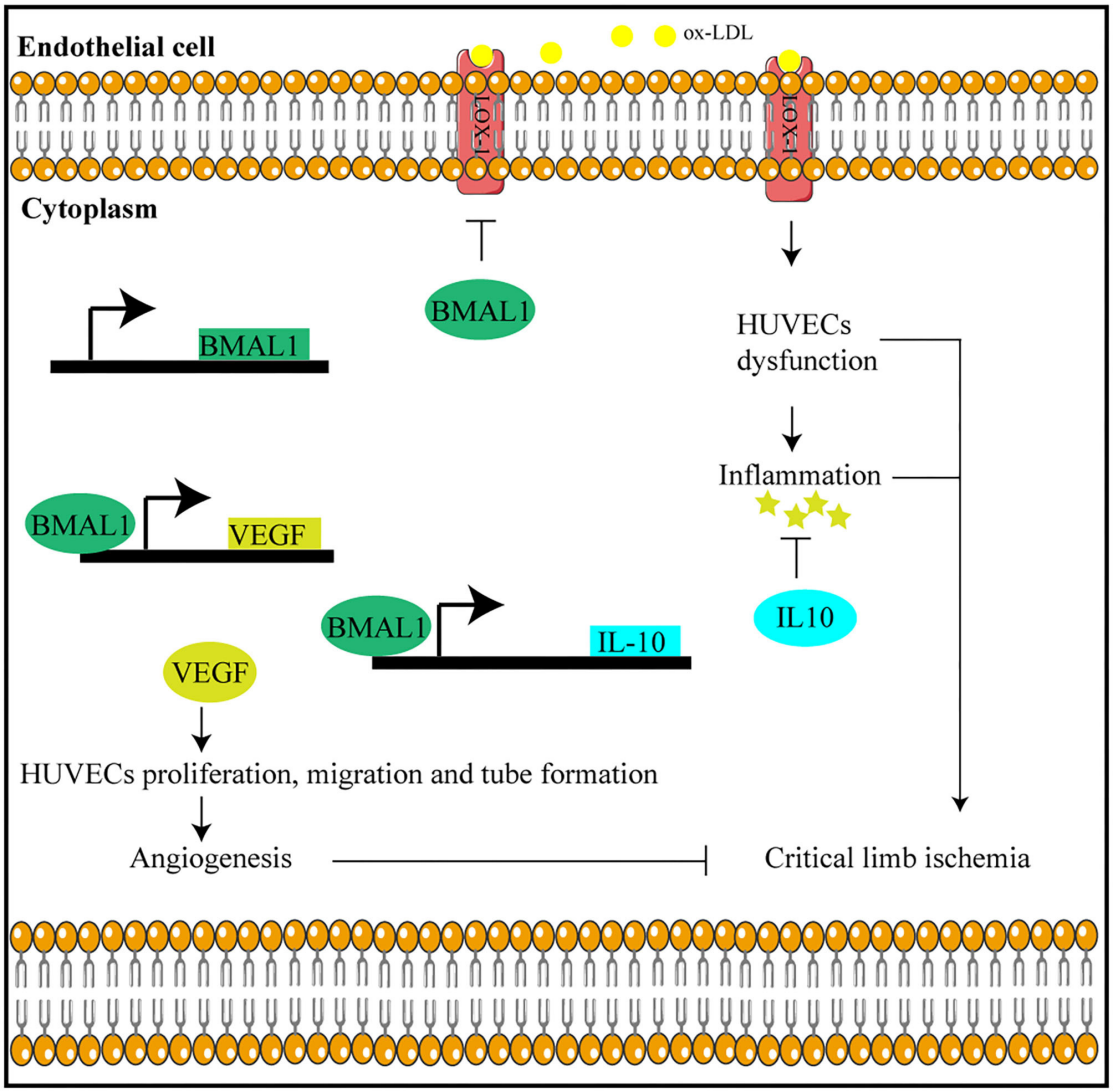
Bmal1 plays a protective role in critical limb ischemia by inhibiting inflammation and promoting angiogenesis.

In this study, we found that circadian gene Bmal1 disruption aggravates critical limb ischemia by promoting lipid uptake and inflammation and impairing angiogenesis. Moreover, Bmal1 transcriptional regulation of VEGF and IL-10 is involved in this process (Figure 8). Thus, targeting therapy of Bmal1 in CLI patients may both promote angiogenesis to recover blood and oxygen supply and inhibit inflammation to alleviate ischemic symptoms.

CLI is the most advanced clinical stage of peripheral arterial disease with high mobility and mortality. It is always associated with atherosclerosis (3). It has been reported that circadian genes are involved in atherosclerosis and cardiovascular diseases. Firstly, global or organ-specific knockout of Bmal1 is reported to attribute to the progression of hyperlipidemia and atherosclerosis (22, 29, 30). Besides this, other circadian genes including Clock (28), Cry (32), and Reverb (33) are all critical in atherosclerosis. However, whether and how circadian clock genes play a role in the occurrence and progression of CLI remains unclear. In this study, we found that the circadian gene Bmal1 plays an important role in CLI. Bmal1 expression in the femoral artery and distal lower limb muscle of CLI patients is decreased. Our study suggested that Bmal1 is a protective factor in CLI. We are the first to indicate the important role of circadian clock in CLI. Therefore, our research suggested that circadian rhythm disorder may be one of the pathogenic factors of CLI. Moreover, our research may provide theoretical basis for CLI treatment, including chronotherapy and gene-based therapy.

Serum lipids have a critical role in the pathogenesis of atherosclerosis and related peripheral vascular diseases. In particular, the serum LDL level is closely related to the human risk of cardiovascular diseases. LDL- and ox-LDL-mediated EC dysfunctions are thought to be the initial step of atherosclerosis (31). The upregulated expression of ox-LDL receptors in the CLI femoral artery and distal lower limb found in our research suggested that excessive lipid uptake and deposition are important factors in the formation and progression of CLI. Moreover, we found that Bmal1 inhibits ox-LDL uptake by repressing the expression of LOX-1, the main ox-LDL receptor of ECs. Thus, attenuated Bmal1 expression in CLI patients leads to increased lipid deposition and severe inflammation response. It has been reported that lipid metabolism is under the regulation of circadian clock. First of all, the concentration of circulating lipids displays a significant circadian rhythm (34), including triglyceride, cholesterol, and LDL (31). Moreover, many genes involved in lipid absorption (35) and biosynthesis (34) are under circadian clock regulation. Consistently, the circadian gene mutant mice, including Clock^Δ19^, Bmal1^−/−^, and Rev-erbα^−/−^ mice, are all hyperlipidemic and prone to cardiovascular diseases (19, 22, 28, 36). The role of Bmal1 in inhibiting LOX-1, CD36, and MSR-1 as found in our research demonstrated that lipid uptake by ECs and macrophages is also under the regulation of the circadian clock. Our research supplements the role of circadian clock in lipid metabolism and cardiovascular diseases.

Emerging evidence suggested that inflammation plays an important role in the progress of CLI (4, 5). In support of this theory, it was shown that the circulating levels of cytokines (IL-6 and TNFα), adhesion molecules (VCAM-1 and ICAM-1), and selectins in patients with peripheral arterial disease are elevated (37). This is consistent with our findings that the pro-inflammatory factor expression is increased in the femoral artery of CLI patients. Moreover, we demonstrated that the expression of inflammatory factors is associated with the severity of ischemic symptoms in the lower limb muscle of CLI patients. Studies have suggested that the circadian clock is involved in the regulation of inflammation. First of all, the number of Ly6C^hi^ monocytes in peripheral blood shows a diurnal oscillation (38), indicating that the circadian clock plays a role in this process. Besides this, it has been demonstrated that Bmal1 represses the expression of Ccl2; thus, myeloid-specific Bmal1 deletion increases monocyte recruitment and worsens atherosclerosis (30). Moreover, Rev-erbα also plays a role in inflammation by regulating the expression of Ccl2 (39). In our research, we verified that circadian gene Bmal1 regulates inflammation by inhibiting lipid uptake and by directly promoting IL-10 expression. Therefore, Bmal1 may inhibit the inflammatory response by promoting the expression of the anti-inflammatory factor IL-10, thereby reducing the ischemic symptoms of CLI.

Angiogenesis occurs in response to tissue hypoxia in CLI patients, and it would lead to feeder collateral and small vessel formation. Angiogenesis plays important roles in many physiological processes, including embryonic development and reproduction (40). Stimulation of angiogenesis can be therapeutic in wound healing and peripheral arterial disease, while excessive angiogenesis may be the basis of certain diseases, including cancer (41) and atherosclerotic plaque vulnerability (42). Controlling angiogenesis is of great value in the treatment of these diseases. Targeting angiogenesis therapy in CLI has attracted great interests these years, mainly by growth factor application and stem cell therapy (1). Despite the application of VEGF (43), FGF (44), HGF (45, 46), and HIF1α (47) in CLI treatment, the effects were minimal mainly because of the formation of immature vessel walls or the activation of inflammation. Among them, only HGF showed a potential therapeutic role in CLI because of its angiogenic property while inhibiting the inflammation function (48). In our research, we found that the circadian gene Bmal1 plays an important role in angiogenesis by transcriptionally regulating VEGF expression. Besides this, it was demonstrated that Bmal1 is involved in the anti-inflammation process by inhibiting lipid uptake and activating IL-10 expression. Therefore, Bmal1 may be a potential therapeutic target in CLI treatment.

In conclusion, we demonstrated that Bmal1 downregulation worsens CLI by impairing angiogenesis and promoting inflammation. Thus, Bmal1 may be a biomarker for diagnosis and a therapeutic target in CLI patients.

## Data Availability Statement

The original contributions presented in the study are included in the article/Supplementary Material, further inquiries can be directed to the corresponding author/s.

## Ethics Statement

The studies involving human participants were reviewed and approved by Ethical Committee of Zhongshan Hospital. The patients/participants provided their written informed consent to participate in this study. The animal study was reviewed and approved by Animal Care and Use Committee of Shanghai Medical College, Fudan University.

## Author Contributions

LX, JJ, DG, and CL conceived the project. LX and CL designed the experiments and wrote and edited the manuscript. LX, QC, YL, YS, and YY performed the experiments. XL and XJ collected the specimen. All authors contributed to the article and approved the submitted version.

## Conflict of Interest

The authors declare that the research was conducted in the absence of any commercial or financial relationships that could be construed as a potential conflict of interest.

## Publisher's Note

All claims expressed in this article are solely those of the authors and do not necessarily represent those of their affiliated organizations, or those of the publisher, the editors and the reviewers. Any product that may be evaluated in this article, or claim that may be made by its manufacturer, is not guaranteed or endorsed by the publisher.
