# Supplementary material for: Bmal1 Downregulation Worsens Critical Limb Ischemia by Promoting Inflammation and Impairing Angiogenesis
Source: Front Cardiovasc Med. 2021 Aug 10;8:712903. doi: 10.3389/fcvm.2021.712903 (PMC8384109; doi:10.3389/fcvm.2021.712903)
Supplement: Supplementary file 1 [file Data_Sheet_1.pdf]

frontiers

# *Supplementary Material*

## Bmal1 downregulation worsens critical limb ischemia by promoting inflammation and impairing angiogenesis

Lirong Xu[1,2†], Yutong Liu [2†], Qianyun Cheng[2†], Yang Shen[3], Ye Yuan[3], Xiaolang Jiang[3], Xu Li[3], Daqiao Guo[3]*, Junhao Jiang [3]*, Changpo Lin [3]*

*Corresponding author:

Changpo Lin: lin.changpo@zs-hospital.sh.cn

Junhao Jiang: jhjiangau@hotmail.com

Daqiao Guo: guo.daqiao@zs-hospital.sh.cn

**This PDF file includes:**

Figure S1 to S6

Table S1 to S4

Figure S1

A

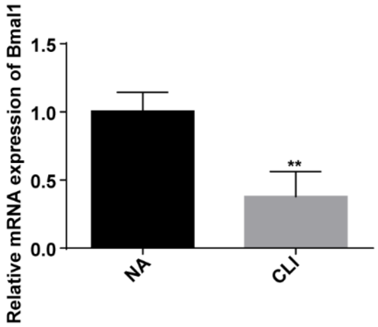


**Figure S1 Bmal1 expression is decreased in the femoral artery of CLI patients**

**(A)** Relative mRNA expression of Bmal1 in the femoral artery of CLI patients and normal artery specimens detected by real-time PCR. Data presented as mean ± SEM (n=3, unpaired t test). **p<0.01 CLI vs NA.

# Figure S2

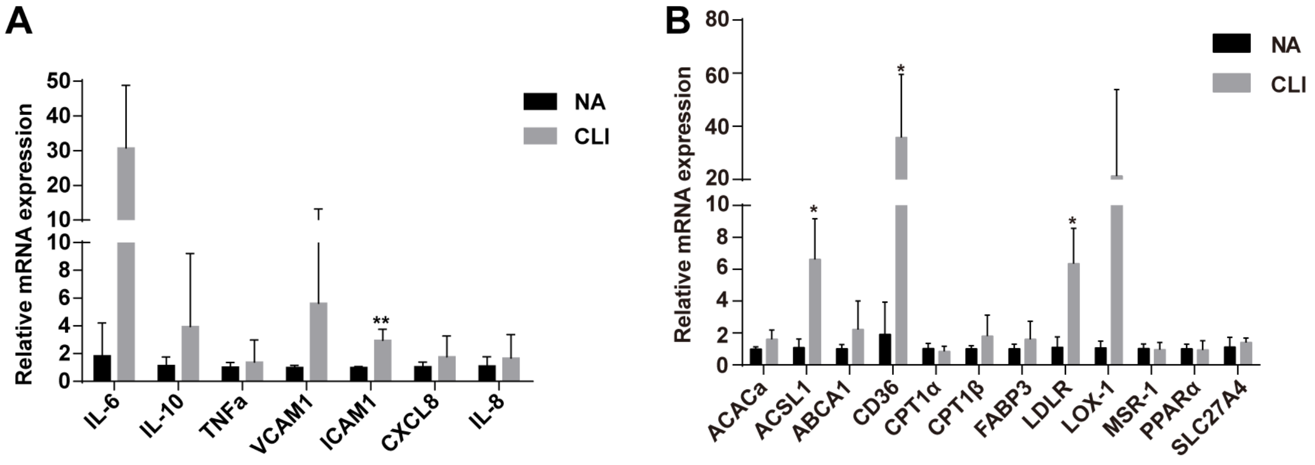


**Figure S2 Expression of genes involved in inflammation and lipids uptake are increased in CLI femoral artery**

**(A-B)** Relative mRNA expression of genes involved in inflammation and lipids metabolism in femoral artery. Data presented as mean ± SEM (n=3, unpaired t test). *p<0.05 and **p<0.01 CLI vs NA.

Figure S3

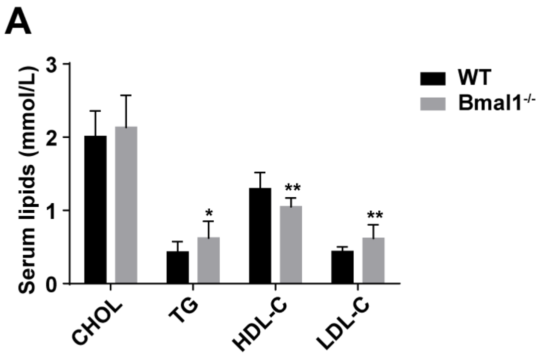


**Figure S3 Serum lipids content of Bmal1$^{-/-}$ mice and their littermates of WT mice**

**(A)** Serum lipids content of Bmal1$^{-/-}$ mice and their littermates of WT mice. Data presented as mean ± SEM (n=5, unpaired t test). *p<0.05 and **p<0.01 Bmal1$^{-/-}$ vs WT.

**Figure S4**

**A**

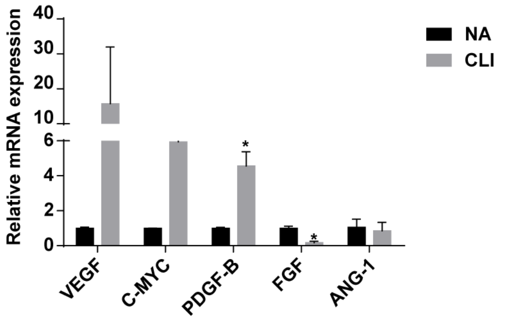


**Figure S4 Angiogenesis is elevated in CLI patients**

**(A)** Relative mRNA expression of genes involved in angiogenesis in the femoral artery of CLI patients and normal artery specimens detected by real-time PCR. Data presented as mean ± SEM (n=3, unpaired t test). *p<0.05 CLI vs NA.

**Figure S5**

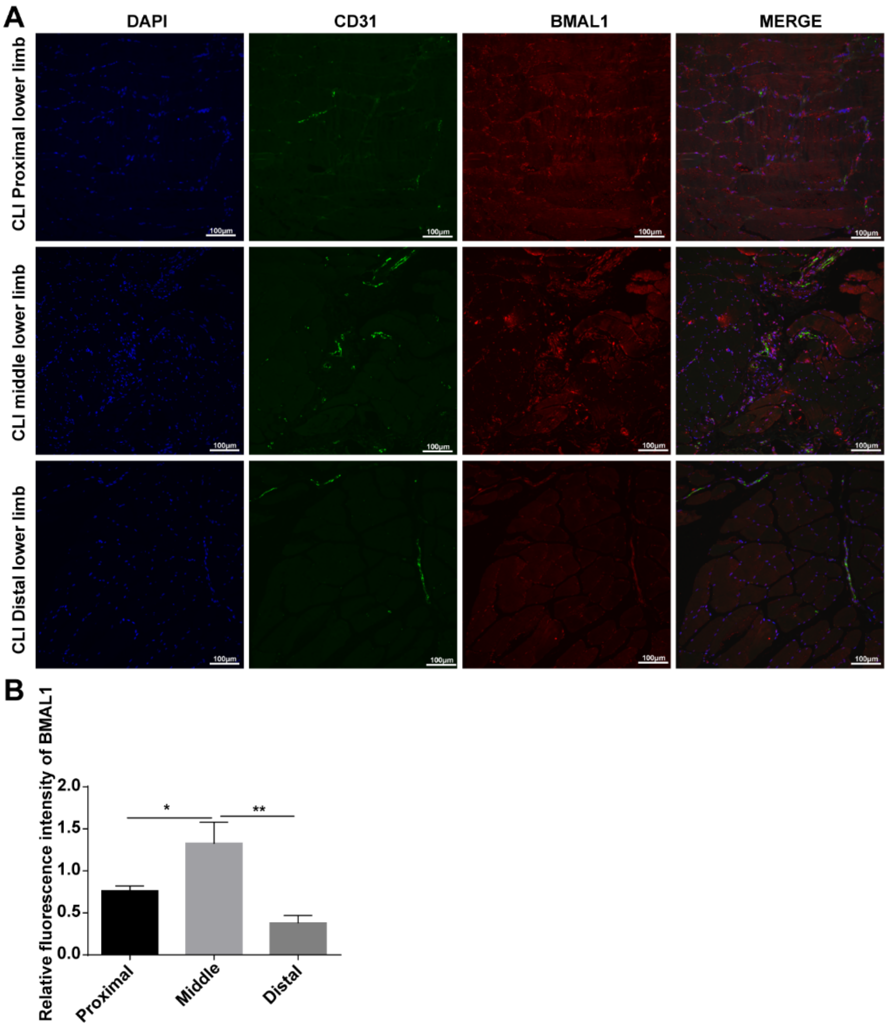


**Figure S5 Bmal1 is down-regulated in the endothelial cells in the distal lower limb muscle of CLI patients.**

**(A-B)** Immunofluorescence co-staining of BMAL1 and CD31 conducted in the lower limb muscle of CLI patients with specific antibodies. Relative fluorescence intensity of BAML1 was determined with image J. Data presented as mean ± SEM (n=9, one way ANOVA with post-hoc Turkey test). *$p<0.05$ middle lower limb vs proximal lower limb; **$p<0.01$ distal lower limb vs middle lower limb.

## Figure S6

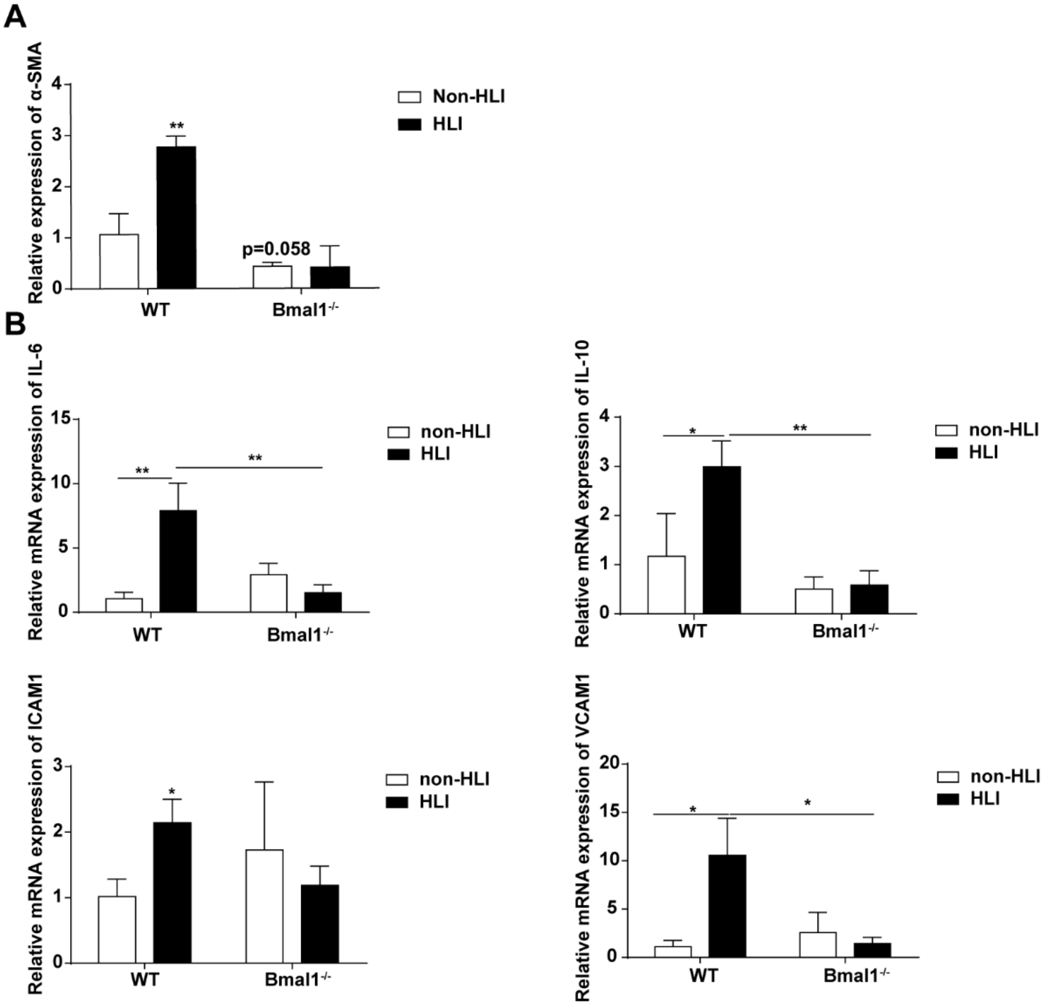


**Figure S6 Angiogenesis is stimulated by HLI in WT mice**

**(A)** Relative mRNA expression of α-SMA in HLI and Non-HLI mice. Data presented as mean ± SEM (n=4, unpaired t test). **p<0.01 WT HLI vs WT Non-HLI.

**(B)** Relative mRNA expression of inflammatory factors in HLI and Non-HLI mice. Data presented as mean ± SEM (n=4, unpaired t test). *p<0.05 and **p<0.01 WT HLI vs WT Non-HLI, *p<0.05 and **p<0.01 Bmal1$^{-/-}$ HLI vs WT HLI.

**Table S1 Primers used in the research**

| | |
|---|---|
| h-GAPDH | Forward: CCATCTTCCAGGAGCGAGATC |
| | Reverse: GCCTTCTCCATGGTGGTGAA |
| h-BMAL1 | Forward: TGGATGAAGACAACGAACCA |
| | Reverse: TAGCTGTTGCCCTCTGGTCT |
| h-VEGF | Forward: ATCTTCAAGCCATCCTGTGTGC |
| | Reverse: CAAGGCCCACAGGGATTTTC |
| h-CD31 | Forward: AACAGTGTTGACATGAAGAGCC |
| | Reverse: TGTAAAACAGCACGTCATCCTT |
| h-c-Myc | Forward: ACAGCGTCTGCTCCACCT |
| | Reverse: CCTCATCTTCTTGTTCCTCCT |
| h-PDGFB | Forward: CTTTAAGAAGGCCACGGTGA |
| | Reverse: CTAGGCTCCAAGGGTCTCCT |
| h-MCP-1 | Forward: CCCCAGTCACCTGCTGTTAT |
| | Reverse: TGGAATCCTGAACCCACTTC |
| h-FGF | Forward: TGCCTCCAGGGAATTACAAG |
| | Reverse: TATAAAAGCCCGTCGGTGTC |
| h-ANG-1 | Forward: AGCGCCGAAGTCCAGAAAAC |
| | Reverse: TACTCTCACGACAGTTGCCAT |
| h-IL-6 | Forward: GCTCCCTACTTCACAAGTCC |

| | |
|---|---|
| | Reverse: GCAGGTTTGCCGAGRAGATC |
| h-IL-10 | Forward: GACTTTAAGGGTTACCTGGGTTG |
| | Reverse: TCACATGCGCCTTGATGTCTG |
| h-TNFα | Forward: CTGGGCAGGTCTACTTTGGG |
| | Reverse: CTGGAGGCCCCAGTTTGAAT |
| h-VCAM1 | Forward: TCAGATTGGAGACTCAGTCATGT |
| | Reverse: ACTCCTCACCTTCCCGCTC |
| h-ICAM1 | Forward: GGCCGGCCAGCTTATACAC |
| | Reverse: TAGACACTTGAGCTCGGGCA |
| h-CXCL8 | Forward: TTTTGCCAAGGAGTGCTAAAGA |
| | Reverse: AACCCTCTGCACCCAGTTTTC |
| h-IL-8 | Forward: CTCTCTTGGCAGCCTTCCTGATT |
| | Reverse: AACTTCTCCACAACCCTCTGCAC |
| h-ACACa | Forward: TCACACCTGAAGACCTTAAAGCC |
| | Reverse: AGCCCACACTGCTTGTACTG |
| h-ACSL1 | Forward: CTTCTGGTACGCCACGAGAC |
| | Reverse: GTCGCTGTCAAGTAGTGCG |
| h-ABCA1 | Forward: ACCCACCCTATGAACAACATGA |
| | Reverse: GAGTCGGGTAACGGAAACAGG |
| h-CD36 | Forward: CGAAAGTCACTGCGACATGA |
| | Reverse: CCTTGGATGGAAGAACGAATC |

| | |
|---|---|
| h-CPT1α | Forward: ATCAATCGGACTCTGGAAACGG |
| | Reverse: TCAGGGAGTAGCGCATGGT |
| h-CPT1β | Forward: GAGGCCTCAATGACCAGAATGT |
| | Reverse: GTGGACTCGCTGGTACAGGAA |
| h-FABP3 | Forward: CACTCACCCACGGCACTGCA |
| | Reverse: TCCCGGTCAGTGGCACCTGA |
| h-LDLR | Forward: ACGGCGTCTCTTCCTATGACA |
| | Reverse: CCCTTGGTATCCGCAACAGA |
| h-LOX-1 | Forward: GCAGAAGAAGCTTCACAGGAGTCAG |
| | Reverse: TGGAGATTCAGATTCTGGTGGTGAAGT |
| h-MSR1 | Forward: GCAGTGGGATCACTTTCACAA |
| | Reverse: AGCTGTCATTGAGCGAGCATC |
| h-PPARα | Forward: ACTTATCCTGTGGTCCCCGG |
| | Reverse: CCGACAGAAAGGCACTTGTGA |
| h-SLC27A4 | Forward: TGGACCCCTCGCTCAGCCTC |
| | Reverse: CAGCCCTGTGGTGCCGGATG |
| m-GAPDH | Forward: TGAAGGTCGGTGTGAACGG |
| | Reverse: CGTGAGTGGAGTCATACTGGAA |
| m-Bmal1 | Forward: GGCTGTTCAGCACATGAAAAC |
| | Reverse: GCTGCCCTGAGAATTAGGTGTT |
| m-Kc | Forward: AGCCACCCGCTCGCTTCTCT |

| | |
|---|---|
| | Reverse: TCGGTTTGGGTGCAGTGGGG |
| m-ACTA2 | Forward: CCCAGACATCAGGGAGTAATGG |
| | Reverse: TCTATCGGATACTTCAGCGTCA |
| m-VEGF | Forward: GCACATAGAGAGAATGAGCTTCC |
| | Reverse: CTCCGCTCTGAACAAGGCT |
| m-CD31 | Forward: GGTGCATGGCGTATCCAAG |
| | Reverse: TGGAGGTCTTATCTATCCTTCGC |
| m-IL-10 | Forward: TCAAACAAAGGACCAGCTGGACAACATACT |
| | Reverse: TCTACCAGGTAAAACTGGATCATTTC |
| m-IL-6 | Forward: GCTTAATTACACATGTTCTCTGGGAAA |
| | Reverse: CAAGTGCATCATCGTTGTTCATAC |
| m-VCAM-1 | Forward: AGTTGGGGATTCGGTTGTTC |
| | Reverse: CATTCCTTACCACCCCATTG |
| m-ICAM-1 | Forward: GTGGCGGGAAAGTTCCTG |
| | Reverse: CGTCTTGCAGGTCATCTTAGGAG |
| m-ACSL1 | Forward: ATCTGGTGGAACGAGGCAAG |
| | Reverse: TCCTTTGGGGTTGCCTGTAG |
| m-CPT1β | Forward: CTGAGACACATCACCGTCTGGAA |
| | Reverse: CACCCCTAAGGATGCCATTCT |
| m-CD36 | Forward: AGATGACGTGGCAAAGAACAG |
| | Reverse: CCTTGGCTAGATAACGAACTCTG |

| | |
|---|---|
| m-FABP3 | Forward: GAA TAG AGT TCG ACG AGG TGA |
| | Reverse: GAA TAG AGT TCG ACG AGG TGA |
| m-LDLR | Forward: CTCCTGCATTCACGGTAGCC |
| | Reverse: CCCACTGTGACACTTGAACTTG |
| m-MSR-1 | Forward: TGAACGAGAGGATGCTGACTG |
| | Reverse: TGTCATTGAACGTGCGTCAAA |
| m-SLC27A4 | Forward: ACTGTTCTCCAAGCTAGTGCT |
| | Reverse: GATGAAGACCCGGATGAAACG |
| VEGF-ChIP- -906/-725 | Forward: AGCTCCACAAACTTGGTGCCAAATT |
| | Reverse: CTGGAGTTGCTTCATGTACAGAGAG |
| VEGF-ChIP- -729/-574 | Forward: TGGGCTCTCTGTACATGAAGCAACT |
| | Reverse: CGAAGACGCTGCTCGCTCCATTCAC |
| VEGF-ChIP- -382/-175 | Forward: CTCTCCGCCTTCCCCTGCCCCCTTC |
| | Reverse: GACGCTCAGTGAAGCCTGGCCCGCA |
| IL10-ChIP- -1986/-1750 | Forward: CTAACACAGACACAGCCCAGAAAAC |
| | Reverse: CAGGGAGTGTCAATGTAGAAACCTA |
| IL10-ChIp- -888/-600 | Forward: AGGTTTCATTCTATGTGCTGGAGAT |

| | |
|---|---|
| | Reverse: CTTTACCCCGATTTCATTAGGATTC |

**Table S2 Antibodies used in the research**

| Target antigen | Vendor or Source | Catalog # | Working concentration |
|---|---|---|---|
| BMAL1 | CST | 14020 | For WB 0.5 μg/ml |
| BMAL1 | Novas | NB100-2288 | For IF 10 μg/ml<br>For ChIP 3μg |
| CD31 | Abcam | ab9498 | For IF 1 μg/ml |
| VEGF | Proteintech | 66828-1-Ig | For WB 0.4 μg/ml |
| GAPDH | Proteintech | 60004-1-Ig | For WB 0.02 μg/ml |
| β-ACTIN | Proteintech | 66009-1-Ig | For WB 0.0427 μg/ml |

**Table S3 primers used for luciferase reporter constructs**

| | |
|---|---|
| hVEGF-2000-luciferase | Forward: ggtaccgagctcttacgcgtgctagcaggaaagttagtggcttccct |
| | Reverse: ccaacagtaccggaatgccaagcttgcggacgctcagtgaagc |
| hVEGF-1216-luciferase | Forward: ggtaccgagctcttacgcgtgctagcgctgatagaagccttggcc |
| hVEGF-971-luciferase | Forward: ggtaccgagctcttacgcgtgctagcctccaacaggtcctcttcc |
| hVEGF-785-luciferase | Forward: ggtaccgagctcttacgcgtgctagcagggctccagatggcacat |
| hVEGF-736-luciferase | Forward: ggtaccgagctcttacgcgtgctagcccagccctgggctctctgtacat |
| hVEGF-540-luciferase | Forward: ggtaccgagctcttacgcgtgctagcgagcgagcagcgtcttcgagagt |
| hVEGF-47-luciferase | Forward: ggtaccgagctcttacgcgtgctagcagtcggctggtagcggggaggatcgc |
| | Reverse: Ccaacagtaccggaatgccaagcttaacccggatcaatgaatatcaa |
| hIL10-2000-luciferase | Forward: ggtaccgagctcttacgcgtgctagcgttagacctgcaggctaacaca |
| | Reverse: Ccaacagtaccggaatgccaagcttagaccttcacctctctgtccc |
| hIL10-1753-luciferase | Forward: ggtaccgagctcttacgcgtgctagcctgaagagttgggaagagacacc |
| hIL10-1185-luciferase | Forward: ggtaccgagctcttacgcgtgctagcgtagagcaacactcctcgccgcaa |
| hIL10-677-luciferase | Forward: ggtaccgagctcttacgcgtgctagcccaagcacagttggggtgggggac |

| | |
|---|---|
| hIL10-324-luciferase | Forward:<br>ggtaccgagctcttacgcgtgctagcaatgagaacccacagctgagggcc |

## Table S4 Characteristics of patients

| CLI patients(n=11) | Mean ± SD or n (%) |
|---|---|
| Age (y; mean ± SD) | 73.36±8.15 |
| Male gender | 8 (72.7) |
| Hypertension | 7 (63.6) |
| Smoker | 3 (27.3) |
| Diabetes mellitus | 6 (54.5） |
| Ischemic heart disease | 4 (36.4) |
| Antiplatelet drugs | 5 (45.5) |
| Lipid-lowering drugs | 3 (27.3) |
| systolic blood pressure (mmHg) | 128±15.40 |
| Total cholesterol (mmol/L) | 3.96±0.66 |

| | |
|---|---|
| HDL cholesterol (mmol/L) | 0.90±0.21 |
| LDL cholesterol (mmol/L) | 2.37±0.59 |
| Triglycerides (mmol/L) | 1.51±0.41 |
| Fasting glucose (mmol/L) | 7.83±2.92 |

HDL, high-density lipoprotein; LDL, low-density lipoprotein
